# Redo-Transcatheter Aortic Valve Replacement Procedural Optimization and Patient Selection: From Bench to Clinical Practice

**DOI:** 10.3390/jcm14082770

**Published:** 2025-04-17

**Authors:** Ruxandra I. Sava, Philippe Garot, Hakim Benamer, Emmanuel Gall, Théo Pezel, Morad Djebbar, Neila Sayah, David Meier, Georgios Tzimas, Jérôme Garot, Florence Leclercq, Mariama Akodad

**Affiliations:** 1 Institut Cardiovasculaire Paris Sud, Hôpital Privé Jacques Cartier, Ramsay Santé, 91300 Massy, France; ruxandra.sava@yahoo.com (R.I.S.); p.garot@angio-icps.com (P.G.); h.benamer@angio-icps.com (H.B.); emmanuel_gall@hotmail.fr (E.G.); moraddjebbar@gmail.com (M.D.); n.sayah@icps.com.fr (N.S.); jgarot@angio-icps.com (J.G.); 2 Department of Cardiology, University Hospital of Lariboisiere, (Assistance Publique des Hôpitaux de Paris, AP-HP), Université Paris-Cité, 75013 Paris, France; theo.pezelccf@gmail.com; 3 Department of Cardiology, Lausanne University Hospital and University of Lausanne, 1005 Lausanne, Switzerland; david.meier1291@gmail.com (D.M.); georgios.tzimas@chuv.ch (G.T.); 4 Montpellier University Hospital, 34295 Montpelier, France; f-leclercq@chu-montpellier.fr

**Keywords:** coronary alignment, coronary access, CT predictors, TAV-in-TAV

## Abstract

With recent guidelines expanding transcatheter aortic valve replacement (TAVR) to younger patients, indications for redo-TAVR will also likely increase. When compared with TAVR, redo-TAVR is a rare and novel procedure. Current clinical data derived from registries suggest excellent safety, with low rates of 30-day and 1-year mortality following redo-TAVR. Proper understanding of data from bench studies regarding optimal valve configurations, of patient anatomy and of the technical properties of transcatheter heart valves (THV) is essential for patient selection and procedural success. Lifetime management of redo-TAVR should start before the index procedure, as the choice of the index THV has a major impact on the feasibility of redo-TAVR. Procedural optimization by adequate valve sizing, commissural alignment and adequate implant depth of both index and redo-THV are critical determinants of optimal hemodynamics for maximized valve longevity, as well as lifelong coronary access.

## 1. Introduction

Aortic valve-in-valve interventions for patients with failed surgical bioprosthetic valves and prohibitive risk for redo surgical aortic valve replacement (redo-SAVR) have been shown to be feasible and safe, with a 30-day mortality rate of 2.5% in recent registries [1]. Following these encouraging results, redo-transcatheter aortic valve replacement (redo-TAVR) was attempted in high-surgical risk patients with a failed initial transcatheter heart valve (THV). As current guidelines support TAVR for younger, lower-risk patients with increased longevity, the indications for redo-TAVR are expected to increase. Studies evaluating patient preferences for TAVR versus SAVR have shown a predilection for minimally invasive procedures [2]. This preference will likely be similar in candidates requiring redo-TAVR, as older patients tend to assign even greater value to quality of life and rapid relief of symptoms over long-term results [2]. However, clinical data on redo-TAVR are scarce and a better understanding of potential issues is crucial to render redo-TAVR feasible in a wider patient population [3]. Lifetime management of aortic stenosis is quintessential, as anticipation of a second or even a third procedure before the first implant is critical to determine the best procedural course in patients with increased life expectancy.

This state-of-the-art review summarizes redo-TAVR bench studies, CT simulation studies and clinical data from patient registries.

## 2. Insights from Bench Studies

Bench studies of various redo-TAVR configurations have offered important preliminary data regarding function, coronary access, valve expansion, embolization risk and leaflet modification techniques. Table 1 summarizes the main findings of THV function in various redo-TAVR configurations.

### 2.1. Leaflet Function

Bench studies have shown that low implant of a short-frame valve within a tall-frame valve is associated with increased *leaflet overhang*, defined as diastolic orifice obstruction caused by inward flexing of the index THV unpinned leaflets. Low implant of the redo-THV may facilitate coronary access, and leaflet overhang has not been shown to acutely impact systolic redo-THV function. However, leaflet overhang may impair diastolic neosinus flow, which may facilitate hypo-attenuated leaflet thickening (HALT) and valve thrombosis [4,5,12]. Moreover, the long-term impact of leaflet overhang in truly failed THVs is unknown, as most bench studies have utilized new THVs with non-calcified leaflets under short-term testing. 

*Leaflet deflection* refers to the distance between the neoskirt (i.e., the deflected leaflets of the index valve) and the index THV frame. Redo-TAVR combinations that do not lead to complete leaflet deflection, such as those utilizing an ACURATE Neo2 (ACn2) valve as the index valve, may carry a lower risk of coronary obstruction [5]. 

*Leaflet pinwheeling* is generally a consequence of valve under-expansion or asymmetric expansion, as the redundant leaflet tissue twists at the free edge. It is more pronounced at low implant depths [13]. Pinwheeling may lead to HALT and early valve degeneration as a result of asymmetrical leaflet shear stress [14,15,16].

### 2.2. Hydrodynamic Performance

Defined by the International Organization for Standardization as a regurgitant fraction (RF) < 20% and transvalvular gradients <20 mmHg [11], hydrodynamic performance was found to be acceptable in most redo-TAVR configurations. Gradients tend to be lower with higher implant positions [4,5,13]. Leaflet overhang has not been found to have a negative impact on transvalvular gradients [4]. However, these studies included only short-term testing and utilized pristine valves as the “failed” THV. This last limitation is crucial, since truly degenerated THVs with thickened leaflets and/or inflow pannus might lead to additional under-expansion of the redo-THV and to leaflet interaction, which may impact long-term THV function to an unknown extent. Of note, a recent study investigating the hydrodynamic function of failed explanted THV valves found that while less than half of the explanted valves were calcified, both groups exhibited increased mean transvalvular gradients [17].

### 2.3. Coronary Access: The Impact of Neoskirt Height

**Figure 1:**
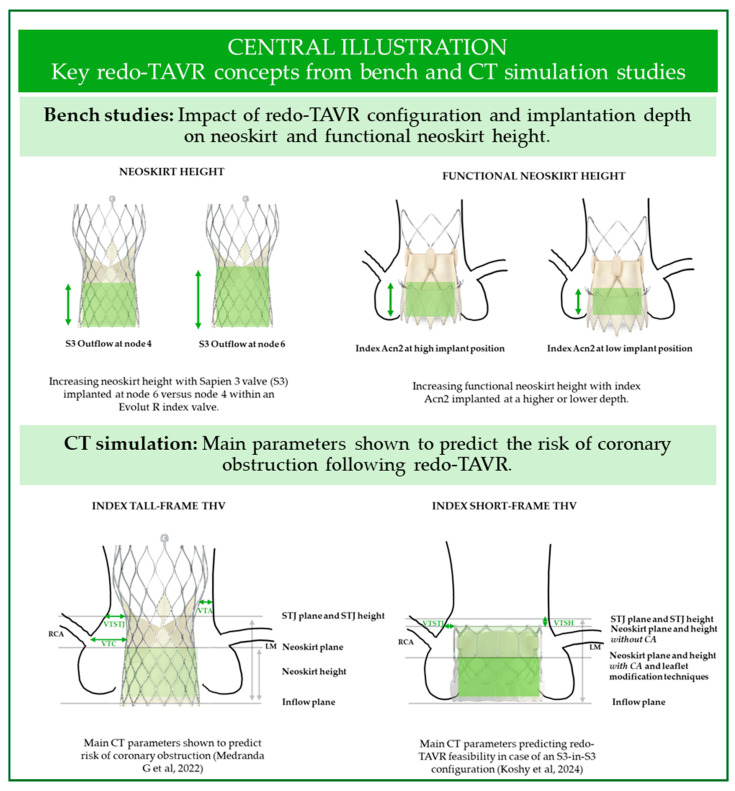
Key concepts from bench and CT studies for redo-TAVR assessment. LM, left main; RCA, right coronary artery; STJ, sinotubular junction; VTA, valve-to-aorta distance; VTC, valve-to-coronary distance; VTSTJ, valve-to-sinotubular-junction distance; VTSH, valve-to-sinotubular junction height [29,32].

In redo-TAVR, the leaflets of the index valve become trapped in an upright position between the index and the redo-THV stent frames. The pinned leaflets create a “graft tube”, also called a *neoskirt*, which is uncrossable with a catheter and does not allow blood flow. In severe circumstances, this may lead to direct coronary obstruction or sinus sequestration and coronary occlusion, especially in case of a taller neoskirts [18,19]. As neoskirt height is defined by the distance between the inflow of the index valve and the level where the index valve leaflets are pinned by the frame of the redo-THV (Figure 1), the height varies according to both index and redo-THV size, design and implantation depth. Specifically, the neoskirt may be shorter when deploying a balloon-expandable valve (BEV) rather than a self-expandable valve (SEV) for redo-TAVR. In bench studies, the lowest neoskirts were achieved with BEV/BEV combination and low implantation of an S3 within an Evolut R [4] or within an Acn2 index valve [5]. The *functional neoskirt* has been defined as the portion of the neoskirt located above the aortic annulus. It is thus dependent on index valve implant depth and is the true determinant of the risk of coronary obstruction as well as the ability to access coronaries. Meticulous pre-TAVR planning is thus essential, especially in case of anatomies at higher risk of coronary obstruction, such as patients with low coronary artery take-off, or those with narrow sinuses of Valsalva and a low sinotubular junction [20].

### 2.4. Coronary Access: The Importance of Commissural Alignment

Commissural alignment (CA) between the THV and the native aortic valve has been shown to facilitate coronary access [21] and reduce the risk of coronary obstruction [22]. It also may contribute to improved leaflet shear stress [23,24]. The impact of CA on HALT and coronary flow is still under investigation. CA can be ascertained on the post-procedural CT scan by measuring the angle between the native commissure and the closest THV commissure. Based on this angle, a THV is considered aligned (0–14°), mildly misaligned (15–29°), moderately misaligned (30° to 44°) or severely misaligned (45° to 60°).

In addition to CA, the size and alignment of THV cells are key determinants of post-procedural coronary access. In a study evaluating redo-TAVR with Sapien, Evolut Pro, ACURATE neo, and Portico valves within Sapien XT or Evolut R THVs, the configuration of ACURATE Neo within Sapien XT resulted in the largest accessible cell, while the configuration of PORTICO within Sapien XT produced the shortest neoskirt. Conversely, Evolut Pro within Evolut R proved to be the least favorable configuration, with the smallest accessible cells and the highest neoskirt from all tested combinations. Moreover, this configuration was more sensitive to cell misalignment, as this reduced cell area by 30–50% compared to when the cells were completely aligned (i.e., both THV frames were perfectly aligned) [9]. A potential limitation of this study is that valve tilting, which was not taken into account, may further hinder coronary access in a clinical setting [25].

## 3. Insights from CT Studies

### 3.1. CT Pre-Procedural Planning: Predicting Coronary Obstruction

Coronary obstruction is a rare (2.5–3.5%) but potentially lethal complication of TAVR in SAVR [26,27,28], associated with around 50% 30-day mortality [28]. CT simulations have been used to predict the risk of coronary flow obstruction and impaired coronary access following valve-in-valve (ViV) and redo-TAVR [29,30]. Key CT measurements used to estimate the risk of coronary flow impairment include the virtual transcatheter heart valve-to-coronary (VTC) distance, valve-to-aorta (VTA) distance at the level of the neoskirt, and the valve-to-sinotubular junction (VT-STJ) (Figure 1). These parameters were shown to be associated with coronary obstruction following ViV TAVR [28]. The main findings of CT simulation studies investigating the feasibility of redo-TAVR are summarized in Table 2.

The accuracy of predicting VTC and VTA measurements using CT-simulated TAVR has been validated against real-life TAVR using paired CT studies completed prior to and 30 days following TAVR implantation [29]. Thus CT-simulated THV implantation may be used to plan the long-term management of younger patients with aortic stenosis, differentiating between anatomies suitable for a redo-TAVR strategy with or without leaflet modification, and those who may require surgical explant of the index THV followed by SAVR [29].

#### 3.1.1. Choice of the Redo-TAVR Valve

Different redo-TAVR configurations are associated with markedly different risks of coronary obstruction and impaired coronary access. In a CT simulation study evaluating the VTC and VTA in redo-TAVR, patients were categorized as being at either *low risk* (VTC ≥ 4 mm and VTA ≥ 2 mm for both coronaries or patients with coronary arteries originating above the neoskirt), at *high risk* (VTC < 4 mm and VTA < 2 mm for both coronaries), or at *intermediate risk* of coronary obstruction (parameters in between). High-risk patients are likely to, and intermediate-risk patients may require leaflet modification to prevent coronary obstruction.

The index THV had a major impact on the risk of coronary flow impairment. While the majority of patients simulated to undergo a redo-TAVR with an index BEV (BEV-in-BEV or SEV-in-BEV TAVR) were predicted to be at low risk (72%), the large majority of those simulated to receive an index SEV (BEV-in-SEV or SEV-in-SEV) were at high risk of coronary obstruction (91% and 75%, respectively) in the absence of leaflet modification techniques [29]. This difference stems from the shorter neoskirt and from the higher proportion of patients with coronary arteries originating above the index BEV. Moreover, leaflet modification techniques such as BASILICA are only an option in the absence of severe commissural misalignment or coronary overlap.

#### 3.1.2. Index Valve Is a SEV

If the index valve is a SEV such as the Evolut, CT simulation studies using data from TAVI patients enrolled in the Evolut Low-Risk CT substudy [30] have shown that choosing a BEV versus a SEV for the second valve is associated with a lower risk of coronary flow impairment. The lowest risk of coronary obstruction was described for S3 valves with the outflow implanted at Evolut node 4 (80% of patients at low risk). Patient-specific factors also played an important role, as female sex, higher BMI, and aortic annulus perimeter-derived diameter < 24.8 mm were independently associated with a higher risk of coronary flow impairment.

The S3-in-Evolut redo-TAVR combination has also been investigated in the context of bicuspid patients. Arslani et al. have recently reported that, in patients previously implanted with an Evolut valve for severe bicuspid aortic disease, redo-TAVR with an S3 THV simulated with the outflow at Evolut nodes 4, 5, and 6 would be unfeasible in 15%, 26% and 38% of patients, respectively [33]. The authors defined unfeasible TAVR as a neoskirt above the coronary risk plane, with a VTC < 4 mm in cases where the neoskirt was below the STJ or with a VTSTJ ≤ 2 mm in cases where the neoskirt was above or at the level of the STJ. These criteria were the same as those used by Grubb et al. when investigating the S3-in-Evolut combination for tricuspid aortic disease. By comparison, the rate of redo-TAVR unfeasibility was considerably higher for every implantation position of the S3-in-Evolut for tricuspid anatomies [30]. Possible reasons for improved feasibility of redo-TAVR in bicuspid anatomies include larger aortic roots, as well as tilting of the index valves away from the coronary arteries by the raphe [33].

Tang et al. have investigated the determinants of coronary access following simulation of S3-in-Evolut redo-TAVR, using data from 219 pre-TAVR CT scans from the Evolut Low-Risk CT substudy and the CoreValve Pivotal trial [31]. Their findings reveal the implantation depth of both index and redo-THV has a major impact on post-procedural coronary access. The highest rate of coronary access was obtained when the index Evolut was simulated at a 5 mm implant depth, with the redo-TAVR S3 outflow being simulated at Evolut node 4 (97%). Implanting the S3 at node 5 significantly reduced coronary access (65%, *p* < 0.001). A higher (3 mm) initial implant depth of the index Evolut was associated with overall lower rates of post-procedural coronary access, which were improved by implantation of the redo-TAVR S3 with its outflow at node 4 (84%) versus implantation at node 5 (31%, *p* < 0.001).

Data on the S3-in-ACn2 redo-TAVR combination have recently been reported by Bieliauskas et al. Using post-procedural CT scans of 153 patients implanted with an ACn2 valve, the authors simulated the S3 position at two implant depths, with low and high implants defined by positioning the S3 outflow at the level of the upper crown or the commissural posts of the ACn2, respectively. Low S3 implantation was associated with a reduced risk of coronary flow compromise, with only 8% of patients being predicted to be at high risk, versus 60% of patients with a high S3 implant. However, the authors found redo-TAVR with this configuration to be feasible in up to 92% of patients [34].

#### 3.1.3. Index Valve Is a BEV

If the index valve is a BEV, the position of the index valve plays an essential role in the feasibility of a future redo-TAVR procedure using a redo-BEV. Using pre-TAVR CT scans from 1900 patients to simulate both the index and the redo-TAVR, Koshy et al. reported a feasibility rate of up to 97.7% if the index S3 was implanted at 80:20 versus 72.5% if the index S3 was implanted at 100:0 (*p* < 0.001). Importantly, the CA of the redo valve, rendering leaflet modification techniques feasible when needed, would result in 100% redo-TAVR feasibility [32].

### 3.2. CT Studies Evaluating Feasibility of Coronary Access in Redo-TAVR Patients

#### 3.2.1. Coronary Artery Origin and the Neoskirt Plane

A multicentric CT study evaluated the feasibility of coronary access in redo-TAVR patients who had been implanted using different redo-TAVR configurations [18]. In this study, the coronary arteries arose above the neoskirt in 10% of patients implanted with an index SEV (CoreValve or Evolut) versus 33% in the case of an initial BEV (Sapien). Parameters of impaired coronary access were a VTA < 3 mm or a distance between stent struts of an overlaid SEV-in-SEV at the level of the crossing zone above the neoskirt < 3 mm, with coronary access being deemed inaccessible when either parameter was <2 mm. The latter measurement is considered the smallest maneuver space required for a 6Fr catheter to be able to engage the coronary artery. Based on these parameters, the investigators reported unfeasible coronary access in 27% of patients implanted with an index SEV, versus only 10% of those implanted with an index BEV.

#### 3.2.2. Commissural Alignment

Initial CT studies following TAVR have shown that THV commissural alignment is usually random, with 22% showing moderate commissural misalignment (CMA) and 31% severe CMA [35]. Misalignment of the Sapien 3 THV has been linked to somewhat worse short-term hemodynamic outcomes in a TAVR population [36]. Indeed, CMA versus CA was associated with a doubling of the frequency of relative mean AV gradient increase over 50% at 30 days [36]. Such an increase has been shown to predict valve thrombosis in SAVR [37], which may in turn promote structural valve degeneration [38].

#### 3.2.3. Commissural Alignment of Self-Expanding Valves

The impact of SEV deployment orientation on post-implant commissural orientation has been studied using co-registration of procedural fluoroscopic views with pre-procedural multislice computed tomography (MSCT). In this pilot imaging study in which MSCT and fluoroscopic images were analyzed retrospectively, tracking the Evolut “hat” marker at the outer curve of the descending aorta, or the ACURATE-neo commissural post at the center back or inner curve, was associated with improved CA and reduced coronary artery overlap. The first prospective, proof-of-concept study to describe a patient and device-specific SEV orientation technique was the COMALIGN study [39]. CA was feasible, achieving at most a mild CMA (<30°) in 53 out of 60 patients (88%), and optimal CA (<15°) in 36 patients on post-procedural MDCT. Moreover, successful alignment of the Evolut THV [40] and the ACURATE neo valve [41] using a similar technique was reported by a second group, with success rates of 95% and 100%, respectively. For more information on commissural alignment techniques, we direct the reader towards another excellent review on the topic [42].

#### 3.2.4. Commissural Alignment of Balloon-Expanding Valves

To date, there is no technique available for commissural alignment of BEVs.

Spilias et al. [43] have described a fluoroscopy-based, semiquantitative method that requires a manual overlay of volume-rendered images derived from pre-TAVR CT onto the fluoroscopic image. This method, which showed excellent correlation with post-TAVR CT imaging (r = 0.93, *p* < 0.01), may allow systematic assessment of CA of BEVs without the need for an additional post-procedural CT.

Akodad et al. [44] proposed a fluoroscopy-based approach that obviates the need for CT overlay. The authors proposed that the offset angle between the posterior THV commissure and the THV center can be calculated using the arcsine function with excellent correlation between the commissural offset angle determined by fluoroscopy and CT (*r* = 0.986). Furthermore, the same group proposed a further simplified method that allows immediate post-procedural ascertainment of CA of a BEV, without the need for additional CT measurements. By using the three-cusp view and the RL-cusp overlap view, patients can be classified on a three-tier scale to define alignment (0–14°), mild to moderate (15–44°), and severe misalignment (45–60°). Using the radio-opaque markers of the BEV, CA was ascertained if the markers were positioned as 1-1-1 on the three-cusp view, and 2-1 in the RL-cusp overlap view. The accuracy of the method was validated against post-procedural CT, showing excellent agreement (weighted kappa coefficient = 0.88) [45].

## 4. Insights from Clinical Studies

Available clinical data on redo-TAVR procedures is scarce and limited to case reports and retrospective registry studies. In the following section, we will summarize registry findings on failure mode, choice of redo-TAVR over TAVR explant and SAVR, and safety and clinical outcomes (Table 3).

### 4.1. Real-World Indications for Redo-TAVR

Redo-TAVR is generally indicated once the initial THV has failed, with criteria for severity being the same as those for native aortic disease. The EXPLANT-OR-REDO-TAVR international registry [47] sought to shed light on real-life indications of redo-TAVR versus surgical explant of the TAVR valve followed by SAVR. Most patients with patient-prosthesis mismatch were treated with a TAVR explant. Structural valve degeneration was a frequent failure mode among both groups of patients but more prevalent in those treated with redo-TAVR. Rates of moderate or severe PV leaks were similar between groups.

### 4.2. Clinical Experience with Redo-TAVR: Success and Failure

The redo-TAVR International Registry [46] included patients implanted with a SEV or a BEV, reporting a success rate of 85.1% according to VARC-2 criteria. Device failure included elevated residual gradients (14.1%) and/or regurgitation (8.9%). The cause of index TAVR failure was considered a consequence of a failed initial procedure in patients presenting within the first year, while primary THV failure was considered the main cause of THV degeneration in patients presenting after one year. The THV failure mode differed between the two groups. Patients with a failed TAVR procedure presented predominantly with valvular regurgitation, while those with a failed THV were equally likely to present with stenosis, regurgitation, or with a combination of both. These findings mirror those reported for TAVR-in-SAVR by the Global Valve-in-Valve Registry [27].

A retrospective analysis of The Redo-TAVR International Registry sought to investigate differences in failure modes between index SEVs and index BEVs, using propensity score matching [3]. When compared to patients receiving an index BEV, patients initially implanted with a SEV presented earlier and more frequently with predominant aortic regurgitation. When investigating redo-TAVR success, the authors found similar results regardless of the index THV, but a higher success rate if the redo-THV was a SEV, as this was associated with lower residual gradients.

### 4.3. Safety and Clinical Outcomes of Redo-TAVR Procedures

While redo-TAVR is a novel procedure, native TAVR is a well-established technique that serves as an adequate comparator for procedural safety. Safety and clinical outcomes of patients from the TVT Registry [49] treated by redo-TAVR with a BEV were compared to those of propensity-matched patients treated by TAVR with a BEV [49]. The authors reported excellent procedural safety, with low complication rates, similar to those recorded in TAVR patients. Specifically, coronary obstruction was reported in four patients (0.3%) and intraprocedural death in eight patients (0.6%). Stroke rates and mortality rates at 30 days and 1 year were all comparable between BEV redo-TAVR and BEV TAVR patients. However, trans-prosthetic gradients were significantly higher in redo-TAVR patients at 1 year (15 mmHg vs. 12 mmHg, *p* < 0.0001), while moderate or severe aortic regurgitation occurred infrequently in both groups (1.8% vs. 3.3%, *p* = 0.8). However, an average 3 mmHg gradient difference, although statistically significant, may not be clinically relevant.

The authors of The Redo-TAVR International Registry [46] also reported good procedural safety among 212 patients, with three strokes (1.4%), seven cases of valve malposition (3.3%), two cases of coronary obstruction (0.9%), and no cases of procedural death. In a separate analysis, these authors compared the safety and procedural success rates between the index and redo-TAVR THVs (BEV versus SEV), using propensity score weighting [3]. Procedural safety and mortality at 30 days were similar between groups, regardless of index or redo-THV. However, the risk of patient–prosthetic mismatch appeared to be reduced by redo-TAVR with a SEV, as residual gradients were lower (10.3 mmHg vs. 15.2 mmHg; *p* < 0.001) in these patients. Rates of moderate or severe regurgitation were similar regardless of the redo-THV type.

In patients who are not candidates for redo-TAVR, a surgical explant of the index THV followed by SAVR is a viable option in patients who are not at prohibitive surgical risk. The EXPLANT-OR-REDO-TAVR International Registry [47] reported that, in 396 patients who underwent either TAVR explant or redo-TAVR, 30-day mortality was lower in patients treated by redo-TAVR (3.4% vs. 13.6%, *p* < 0.001). Similarly, fewer deaths were recorded in the redo-TAVR group at 1 year. However, this difference was largely determined by the initially high 30-day mortality rate that included in-hospital mortality, as a landmark analysis performed at 30 days showed similar mortality between groups between the 30-day and 1-year mark (*p* = 0.91).

Moreover, the investigator-initiated ReTAVI registry will collect prospective procedural and outcome data of patients undergoing a redo-TAVR with the Sapien BEV in 60 high-volume centers across Europe, with a pre-specified 30-day and 12-month follow-up [50].

## 5. Conclusions and Future Directions

While redo-TAVR is a novel procedure, registry safety data are reassuring, with similar 30-day and 1-year mortality when compared to native-TAVR, and markedly reduced 30-day and 1-year mortality when compared to TAVR explant. However, data from bench and CT studies suggest that meticulous patient selection and pre-procedural planning are key determinants of procedural safety. A lifetime management strategy needs to be considered when planning the initial THV procedure, as index THV type and implantation depth are critical parameters of redo-TAVR feasibility.

Thanks to their lower frame height and hence reduced neoskirt, BEVs are likely an essential component of redo-TAVR procedures, either as an index or as a redo-THV. In this clinical context, CA of BEVs is of paramount importance to mitigate the risk of coronary obstruction and ensure lifelong coronary access, with CT simulation studies suggesting that leaflet modification techniques may render redo-TAVR feasible in the vast majority of patients with a S3-in-S3 configuration. The next-generation Sapien valve is designed to facilitate CA and will likely expand TAVR indications in patients with more challenging anatomies as well as in the younger, active population who values quality of life and desires to avoid the need for long-term anticoagulation associated with mechanical heart valves. SEV valves may also serve as optimal redo-THVs, as they ensure lower residual gradients.

There are currently no specific manufacturer recommendations for sizing of redo-THVs, given the wide range of possible combinations. Nevertheless, a sizing algorithm has been recently proposed based on experts’ consensus [51]. Moreover, as redo-TAVR is a relatively novel procedure, there are no robust data regarding the durability of a second TAVR implant. Adequate sizing is likely essential to optimize valve durability, and 3D printing and computational modeling may offer future solutions for enhanced patient personalization and optimal hemodynamics.

Finally, most studies have included highly selected patients, and there are a lack of data on patients requiring a redo-TAVR procedure but who were ineligible due to anatomical factors. Investigating redo-TAVR outcomes after stratifying based on pre-procedural CT evaluation of anatomic risk factors may offer insight into the outcomes of patients with non-ideal anatomy for redo-TAVR.

## Figures and Tables

**Table 1:** Summary of bench studies evaluating different redo-TAVR configurations, their main outcomes and results.

Study	Redo-TAVR Valve Types	Specific Valves	Design	Main Outcomes	Results
Akodad M. et al. [4]	BEV in SEV	Sapien 3 (S3, 20–29 mm) in Evolut (23–34 mm)	S3 outflow implanted at Evolut nodes 4, 5 and 6	1. Neoskirt height	Shortest at node 4, highest at node 6
				2. Leaflet overhang	Greatest when low implantation depth
				3. Hydrodynamic performance *	Acceptable RF < 20% for all except 29 mm S3 at node 4 of 29 mm Evolut
				4. Embolization risk	Low at all implant depths
Akodad M. et al. [5]	BEV in SEV	Sapien 3 Ultra (S3U) in ACURATE neo2 (ACn2)	*Low* implant (S3U outflow at ACn2 upper crown) vs. *high* implant (S3U outflow at base of ACn2 commissural post)	1. Neoskirt height	Shorter when S3U at low implant depth
				2. Leaflet overhang	Moderate< 50% for all except 26 mm S3U implanted low in L ACn2
				3. Leaflet deflection	>2 mm gap between neoskirt–outer border of THV frame
				4. Valve expansion	S3U under-expanded, 78–92% of expected nominal area
				5. Hydrodynamic performance *	Favorable in all configurations
Akodad M. et al. [6]	SEV (ALLEGRA 27 mm) in various SEV and BEV	ALLEGRA in: Evolut Pro (EvP, 26 mm) Lotus (25 mm) JenaValve (25 mm) Portico (25 mm) S3 (23 mm) ALLEGRA (27 mm) Acn (M size)	Each configuration at −4, 0, and 4 mm implantation depth (distance from lower border of redo valve to lower border of index valve)	1. Hydrodynamic performance	All configurations were compatible, except ALLEGRA in EvP at −4 mm (outflow constrained by Evolut Pro waist)
				2. Transvalvular gradients	<20 mmHg in all compatible configurations
				3. Pinwheeling	In EvP: more important irrespective of implantation depth In the other THVs: worse at high implantation depth
				4. Neoskirt height	Higher for tall frame THVs
Sathananthan J. et al. [7]	SEV/BEV in SEV/BEV	S3, Evolut Pro, Acn, ALLEGRA, Portico in Sapien XT (SXT) and Evolut R	Sapien XT (23-29 mm) Evolut R (23-29 mm)	1. Anchoring	Most stable S3 in an Evolut R requires adequate sizing to prevent embolization; ALLEGRA and Portico embolized from 29 mm SXT
				2. Hydrodynamic performance	Acceptable for all valves implanted within the SXT; Improved for Evolut R, Acn implanted high; High RF if S3 implanted low in Evolut R
				3. Transvalvular gradients	Acceptable
Meier D. et al. [8]	BEV in BEV	S3 (23 mm) in SX (23 mm) or S3 (23 mm)	Evaluation of index and redo valve expansion and hydrodynamic performance without and with pre and/or post-dilation (23 mm non-compliant balloon)	1. Expansion without pre/post-dilation:	S3 under-expanded
				2. Pre and post-dilation	Redo S3 remained under-expanded when implanted in an SXT
					Redo S3 achieved nominal diameter when implanted in an S3
					Index valve was overexpanded (12%)
				3. Hydrodynamic performance	Acceptable
					Increased pinwheeling in under-expanded valves
Meier D. et al. [9]	SEV/BEV in BEV	S3, Evolut Pro, Acn, Portico, in SXT, and Evolut R	Micro-computed tomography determination of neoskirt height and size lowest accessible cell for coronary access	1. Shorter neoskirts	Most cases with index SXT Shortest: Portico in SXT
				2. Higher neoskirts	Various configurations within EvolutR Highest: high implant of 26 mm Evolut Pro in 25 mm Evolut R
				3. Largest accessible cell	Acn in SXT
				4. Smallest accessible cell	Evolut Pro in Evolut R; Misalignment in this configuration reduced cell area by 30–50%
Meier D. et al. [10]	SEV in BEV	Acn2 or Ac XL in S3 vs. S3 in S3	BEV implanted nominally low or nominally high	1. Hydrodynamic performance	More favorable for Acn2/Ac XL in S3 Implantation height had minimal impact
				2. Pinwheeling	Less for Acn2/Ac XL in S3
				3. Neoskirt length	Slightly taller for Acn2/Ac XL

Acn, ACURATE neo; Acn2, ACURATE neo2; Ac XL, ACURATE Prime XL; BEV, balloon-expandable valve; S3, Sapien 3; S3U, Sapien 3 Ultra; SXT, Sapient XT. * Acceptable according to ISO 5840-3 guidelines [11].

**Table 2:** Redo-TAVR CT simulation studies and the main parameters shown to predict coronary obstruction after redo-TAVR.

Author	Study Design	Main Outcomes	Results
Medranda GA et al. [29]	CT redo-TAVR simulation using paired, pre-, and post-TAVR CT studies N = 213	Patients classified into Low risk of coronary obstruction: VTC ≥ 4 mm and VTA ≥ 2 mm for both coronaries Or both coronaries above pinned leaflet plane High risk of coronary obstruction: VTC < 4 mm and VTA < 2 mm for both coronaries	Patients predicted to be at low risk: 25.4%
			Patients predicted to be at high risk and likely requiring leaflet modification: 27.7%
			Redo-TAVR feasible only if first valve a BEV and likely requiring leaflet modification: 46.9%
Grubb KJ et al. [30]	Post-TAVR CT scans N = 204	Evaluation of five redo-TAVR implant depths: S3-in-Evolut inflow-to-inflow, S3 outflow at Evolut nodes 4, 5, 6, Evolut-in-Evolut inflow-to-inflow	Lowest risk of coronary obstruction: S3 outflow at Evolut node 4
			Highest risk of coronary obstruction: S3 outflow at Evolut node 6 Evolut-in-Evolut
Tang GHL [31]	CT simulation of index (Evolut) and redo-TAVR (S3) valves N = 219	Impact of implantation depth of index and redo-TAVR on coronary access	Highest rate of coronary access (97%): Index: Evolut implanted at 5 mm depth Redo-TAVR: S3 implanted with outflow at Evolut node 4
			Lowest rate of coronary access (31%): Index: Evolut implanted at 3 mm depth Redo-TAVR: S3 implanted with outflow at Evolut node 5
Koshy AN [32]	CT simulation of index (S3) and redo-TAVR (S3) valves N = 1900	Impact of implantation depth of index THV in an S3-in-S3 simulation study	Reduced redo-TAVR unfeasibility in case of higher index implant position 100:0 vs. 80:20–redo-TAVR unfeasible in 27.5% vs. 2.3% (*p* < 0.01).
			CA associated with redo-TAVR feasibility in all patients

**Table 3:** Main outcomes reported by registry studies including patients treated by redo-TAVR.

Registry	Study Design	Main Outcomes	Results
**Redo-TAVR** **International ** **Registry** [46]	Investigator-initiated, international, 37 centers Core lab assessment for baseline echocardiography and CT N = 212	30-day mortality: Failed TAVR procedure Failed TAVR valve	5.4% 1.3%
		THV failure mode if:	
		Failed TAVR procedure	Valvular regurgitation: 73% Valve stenosis: 16% Mixed: 11%
		Failed TAVR valve	Valvular regurgitation: 30% Valve stenosis: 37% Mixed: 33%
**Retrospective ** **analysis of ** **Redo-TAVR ** **International ** **Registry** [3]	Propensity score matching according to index or redo SEV or BEV N = 221	Index valve type	If index valve is SEV
		THV failure presentation	Earlier failure: 3.7 ± 2.3 years vs. 4.9 ± 2.1 years; *p* < 0.001
		THV failure mode	More frequently with AR: 47.3% vs. 16.2%; *p* < 0.001
		Redo-TAVR success	
		Index valve type	Similar
		Redo valve type	Higher in case of redo-SEV: 77.2% vs. 64.3%; *p* = 0.045 Lower gradients with redo-SEV: 10.3 mmHg vs. 15.2 mmHg; *p* < 0.001
**EXPLANT-OR-REDO-TAVR international registry** [47]	International registry, 29 centers Patients with failed TAVR treated by surgical explant vs. redo-TAVR N = 396	Treatment based on index valve failure mode:	
		Patient-prosthesis mismatch	More frequently by TAVR explant: 17.1% vs. 0.5%; *p* < 0.001
		Structural valve degeneration	More frequently by redo-TAVR: 63.7% vs. 51.9%; *p* = 0.023
		Mortality	
		At 30 days	Lower for redo-TAVR 3.4% vs. 13.6%, *p* < 0.001
		At 1 year	Lower for redo-TAVR 15.4% vs. 32.4%, *p* = 0.001
**TVT registry** [48]	Multicenter registry based in the USA Propensity score matching of 1320 BEV redo-TAVR patients with 1320 BEV TAVR patients	Stroke at 30 days	Similar 2% vs. 1.9%, *p* = 0.84
		Stroke at 1 year	Similar 3.2% vs. 3.5%, *p* = 0.80
		Mortality at 30 days	Similar 4.7% vs. 4.0%, *p* = 0.36
		Mortality at 1 year	Similar 17.5% vs. 19.0%, *p* = 0.57

## Abbreviations

BEV	Balloon-expandable valve
CA	Commissural alignment
CMA	Commissural misalignment
CT	Computed tomography
HALT	Hypo-attenuated leaflet thickening
TAVR	Transcatheter aortic valve replacement
THV	Transcatheter heart valve
SAVR	Surgical aortic valve replacement
SEV	Self-expandable valve
VTA	Valve-to-aorta distance
VTC	Valve-to-coronary distance
